# Dataset of microscope images of prefrontal cortex from wistar rat tissue after an induced stroke for image registration and stitching

**DOI:** 10.1016/j.dib.2021.107066

**Published:** 2021-04-21

**Authors:** Bladimir. Salas-Quinchucua, Jean P. Díaz-Paz, Humberto Loaiza-Correa, John Umbarila-Prieto

**Affiliations:** a Escuela de Ingeniería Eléctrica y Electrónica, Universidad del Valle, Cali, VA, 760032, Colombia; b Centro de Investigación, Innovación y Desarrollo. CIID - FUNDACIÓN FICC, Colombia

**Keywords:** Image registration, Stitching, High-resolution image, Prefrontal cortex tissue, Homography

## Abstract

This article presents a dataset of raw microscopic images of the prefrontal cortex from wistar rat tissues, after an induced stroke, stained with NeuN antibody. The raw images were captured using a microscope equipped with a digital camera. The dataset is useful for testing techniques for the improvement, registration, and stitching to generate a high-resolution image with a full reconstruction of tissues. Besides, this dataset can be used to assess the neuronal brain after an ischemic event. The dataset contains 1370 microscope images with 20x magnification and 36 (*Hierarchical Data Format version 5)* hdf5 files with homography matrices between every pair of sequential images per tissue rows.

**Specifications Table**

**Table:** 

Subject	Computer Vision and Pattern Recognition
Specific subject area	Microscope images registration and stitching
Type of data	Images, HDF5 files, and one high-resolution stitched image
How data were Captured	*Leica DM500 microscope*^1^, Leica ICC50HD camera^2^
Data format	Raw Microscope images in PNG format (1920×1080) format. High-resolution stitched image in PNG format (42300×24885), pixel size 0.3 µm, area 92.48 mm^2^. Homography matrices HDF5 files
Parameters for data collection	Microscope´s magnification (20x). Horizontal (56%) and vertical (40%) overlap for image capture
Description of data collection	Raw microscopic images were captured with the Leica ICC50HD camera mounted on the Leica DM500 microscope, performed with fixed parameters in magnification, and horizontal and vertical overlap*.* The high-resolution image was generated using the captured images and a developed algorithm. The homography matrices were obtained from a small number of correspondences manually selected between each pair of images horizontal displacements and between pair of rows in vertical displacement.
Data source location	Institution: Universidad del Valle. City/Town/Region: Cali / Valle del Cauca Country: Colombia
Data accessibility	Repository name: Mendeley Data Data identification number: http://dx.doi.org/10.17632/9t4246w5sw.1 Direct URL to data: http://dx.doi.org/10.17632/9t4246w5sw.2

## Value of the Data

• This dataset presents microscope images of microscope capture and the homography matrices between each pair of images. These data are useful to evaluate and develop computer vision algorithms as register and stitching algorithms.
• The dataset can be used in pattern recognition and computer vision researchers to develop, improve, and test methods for detection, description, matching or segmentation techniques.
• The homography matrices included in this dataset can be used to evaluate errors in registration processes focused on microscope images.
• Medical researchers can be allowed to evaluate NeuN immunoreactive changes that may indicate changes in connectivity in any area of the brain, including injured focus, exofocal or any area in the high-resolution image of the whole tissue (Fig. 3).

## 1 Data Description

**Fig. 1:**
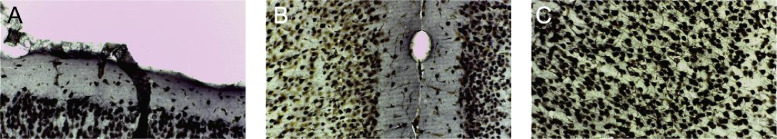
Three images of the dataset.

**Fig. 2:**
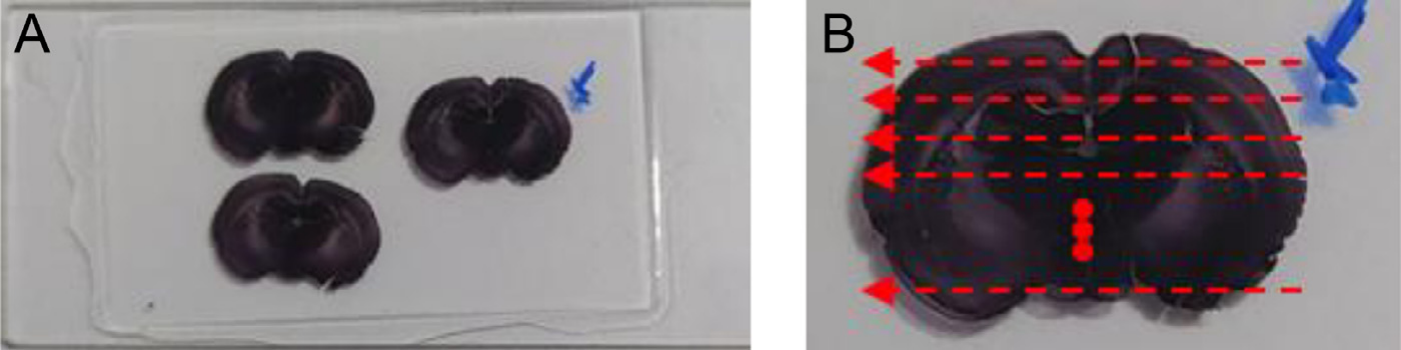
Microscope images capture protocol. The red lines illustrate microscope displacement in capture protocol.

**Fig. 3:**
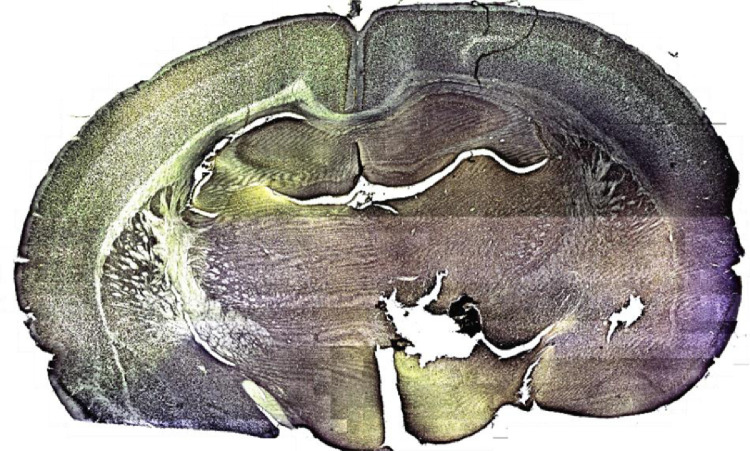
High-Resolution Image of the whole tissue. File size: 1,3GB, For full resolution please download at dataset Mendeley files in /stitched/tissue/ brainTissueImage.png, Dataset URL: http://dx.doi.org/10.17632/9t4246w5sw.2, Image download URL: https://data.mendeley.com/datasets/9t4246w5sw/2/files/06126c1c-83fe-419f-8ef9–027c5535cbcd.

**Diagram 1:**
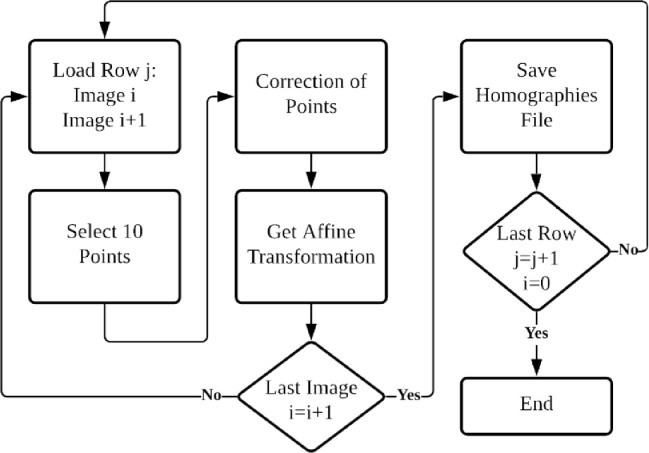
Write the homographies matrix between images.

The dataset contains sequential raw images of the prefrontal cortex from rat tissue stained with NeuN antibody tissue in glass slides. There are 36 rows of images captured from right to the left direction, as shown in Fig. 2, using an electromechanical stage with 20x magnification and are compressed in PNG format. Also, the dataset includes one high-resolution image (41,300 × 24,885), one folder with the homography matrices between each horizontal sequential pair of images, and between rows. Table 1 describes the organization of the dataset folders and the names of their files; Table 2 presents the main specifications of the camera used to build the image data set; Table 3 presents the main specifications of the microscope used to capture the images. Fig. 1 shows examples of microscope images from the data set; Fig. 2 describes the path followed to take the images from the microscope, and Fig. 3 shows the high-resolution stitched image obtained. Diagram 1 shows the diagram algorithm used to write the homographies between the images.

**Table 1:** Dataset organization.

Folder	Filename	Description
\stitched\tissue\	brainTissueImage.png	High-resolution obtained.
\stitched\rows	image_Row_XX.png	Images for each row numbered as RR (01–36).
\homographies\	homography_images_Row_RR.h5 homography_Rows.h5	Homography matrices between each pair of images per row numbered as RR (01–36).
\rawImages\	image_Row_RR_XX.png	Raw images of tissue numbered as XX (varies each row: 01 up to 45) of images rows numbered as RR (01–36).

**Table 2:** Cameras specifications.

Model	Image size (pixels)	Sensor size
ICC50HD	1920 × 1080	6.55 mm × 4.92mm

**Table 3:** Microscope Leica DM500 specifications.

Reference	Type	Magnification	Light	Specimen
Leica DM500	Compound	20x	Incident, dimmable	glass slides

## 2 Experimental Design, Materials and Methods

### 2.1 Tissue

Twenty-eight adult, male, Wistar rats were subjected to focal injury in the right hemisphere. The specimens weighted between 240 and 320 g (average 280 g), and were aged between 120 and 180 days. It was followed the protocol to avoid unnecessary suffering.

The technique used was the experimental model of focal ischemic injury through intraluminal suture of the middle cerebral artery. Analyses were made for the five groups: and after the lesion (control), at 24 h, 96 h, 10 days, and 20 days.

At the corresponding time, the specimens of each group were anesthetized and transcardially perfused through the left ventricle with 200 mL of 0.9% saline, followed by 200 mL of a mixture of paraformaldehyde (4.0%), lysine and sodium periodate in 0.1 M phosphate buffer (pH 7.4). Then, the brains were removed from the braincase and left in the fixative. Later, 10 coronal sections of 30 μm thickness were performed in the vibratome of liquid medium. The sections were incubated with the primary antibody NeuN.

Exofocal neuronal damage was inferred from neuronal immunoreactivity changes to NeuN.

### 2.2 Hardware and Software

For capturing the images of the database, the next materials and equipment were used:

• Leica ICC50HD (Specifications in Table 2).
• Leica Microscope DM500 (Specifications in Table 3)
• Python Capture Application.

Microscope images were captured with an approximate horizontal overlap of 56% (~0.321 mm between consecutive images) and a vertical overlap of 36% (~0.12 mm between consecutive rows) using a Leica ICC50HD camera and DM500 microscope with 20x magnification. Fig. 1 shows three microscope images that were captured from glass slides of wistar rat's tissue 20 days before an induced stroke (glass slides are property of “*Grupo de investigación: Bienestar, trabajo, cultura y Sociedad. Pontificia Universidad Javeriana Cali”* which perform the animal experiment, they allowed us through doctor Umbarila, author in this work and previous [1] to capture the images dataset to use for this work., due to that, the microscope images captured in this work can be distributed freely.

The capture protocol consisted of moving the slide, as shown in Fig. 2. The displacement was executed by a computer interface and electromechanical 3D printed device attached to the microscope stage. The images were captured only when the tissue was present, so each row has a different quantity of images (between 11 and 45). The capture image process starts at a home position (top-right of the tissue). Then, controlled displacements are performed from right to left holding for every capture until there is no tissue on the captured image. After, the stage returns to the first position of the row and performs a vertical shift. The controlled displacements are repeated until the whole tissue is covered (36 rows).

The homography matrices were calculated between every consecutive pair of images per row as affine transformations. The homographies were computed using Diagram 1, adapted from [2], in which at least 10 points between two images (reference and target images) were manually marked. Then, the selected points are tuned using cross-correlation. After, it calculates the homography matrix approximating an affine transformation from points. The obtained homographies are compatible with the well-known assessment protocol for detection and description that was proposed by [4] and has been used to create microscopic images mosaicking with and automatic method based on SIFT feature detection [3]. Or a comparison between feature detection using SIFT, SURF, Harris, and ORB algorithms by [5].

## Ethics Statement

This work does not perform any human or animal experiment, however, images captured from glass slides of tissue from the experiment perform by [1], where declared that *“The experimental protocol was assessed and approved by the ethics committee of the Universidad Libre (Cali, Colombia), to comply with the current regulations of the European Economic Community for the use and care of animals used for experimental and other scientific purposes (Strasbourg, 15 June of 2006), and the Colombian law (Law 84 of 1989 and Resolution No. 8430 of the Ministry of Health, 1993) on ethics, care and control of animals for experimental purposes”.*

## CRediT Author Statement

**Bladimir Salas-Quinchucua:** Conceptualization, Methodology, investigation, Software; Writing - Original draft preparation, Data curation; **Jean P. Díaz-Paz:** Conceptualization, Software, Data curation; **Humberto Loaiza-Correa:** Supervision, Reviewing, Conceptualization, Reviewing and Editing; **John Umbarila-Prieto:** Data Curation (glass slides), Validation, Reviewing and Editing.

## Declaration of Competing Interest

The authors declare that they have no known competing financial interests or personal relationships which have or could be perceived to have influenced the work reported in this article.

## Appendix Supplementary Materials

Image, application 1

## Footnotes

1 https://www.leica-microsystems.com/products/light-microscopes/p/leica-dm500/.

2 https://www.leica-microsystems.com/products/microscope-cameras/p/leica-icc50-hd/downloads/.
