# Supplementary material for: Dataset of microscope images of prefrontal cortex from wistar rat tissue after an induced stroke for image registration and stitching
Source: Data Brief. 2021 Apr 21;36:107066. doi: 10.1016/j.dib.2021.107066 (PMC8114116; doi:10.1016/j.dib.2021.107066)
Supplement: Supplementary file 1 [file mmc1.pdf]

Colombia Médica
colombiamedica.univalle.edu.co

Original Article

Assessment transcallosal Diaschisis in a model of focal cerebral ischemia in rats

Evaluación de la diásquisis transcallosa en un modelo de isquemia cerebral focal en ratas

César Augusto Arango-Dávila[1,2], Beatriz Elena Muñoz Ospina[3], Daniel Manrique Castaño[3,4], Laura Potes[1,2], John Umbarila Prieto[4].

[1] Fundación Valle del Lili, Cali, Colombia.
[2] Biomedical Research Group, Universidad ICESI, Cali, Colombia.
[3] Psychological Assessment and Measurement Group, Pontificia Universidad Javeriana, Cali, Colombia.
[4] Center for Research, Innovation and Development, CIID . Fundación Innovación y Ciencia por Colombia. FICC, Bogotá, Colombia.

Arango-Dávila CA, Muñoz OBE, Manrique C D, Potes L, Umbarila PJ. Assessment transcallosal Diaschisis in a model of focal cerebral ischemia in rats. Colomb Med (Cali). 2016; 47(2): 87-93.

© 2016. Universidad del Valle. This is an Open Access article distributed under the terms of the Creative Commons Attribution License, which permits unrestricted use, distribution, and reproduction in any medium, provided the original author and source are credited.

Article history:

Received:05 November 2015
Revised: 26 April 2016
Accepte: 19 May 2016

Keywords:

Diaschisis, brain ischemia, immunohistochemistry, physiological stress, brain Injuries, apoptosis.

Palabras clave:

Isquemia cerebral, Inmunoistoquímica, lesión cerebral, estrés fisiológico, apoptosis.

Abstract

Objective: To evaluate transcallosal changes after a local ischemic injury in rats by using the monoclonal marker anti-NeuN (Mouse anti-neuronal nuclei).

Methods: Twenty eight adult, male, Wistar rats were subjected to focal injury in the right hemisphere. The technique used was the experimental model of focal ischemic injury through intraluminal suture of the middle cerebral artery. Analyses were made for the five groups: and after the lesion (control), at 24 h, 96 h, 10 days and 20 days. Exofocal neuronal damage was inferred from neuronal immunoreactivity changes to NeuN.

Results: In the cortex contralateral to the lesion, immunoreactivity was diminished. This was most notable in the supragranular layers 24 h post ischemia. After 96 h, there was a generalized diminishment of the inmmunoreactivity in supra and infragranular layers. At 10 and 20 days, the tissue recovered some NeuN immunoreactivity, but there were set changes in the VI layer.

Conclusion: The immunoreactive changes to NeuN support the process of interhemispheric diaschisis. Changes in immunoreactivity could indicate metabolic stress secondary to the disruption in connectivity to the site of lesion.

Resumen

Objetivo: Evaluar los cambios exofocales transcallosos después de lesión isquémica focal en ratas, mediante marcación inmunohistoquímica con el anticuerpo monoclonal anti-NeuN (Mouse Anti-Neuronal Nuclei).

Métodos: Se intervinieron 28 ratas machos Wistar adultas. Mediante el modelo experimental de isquemia cerebral focal del territorio de la arteria cerebral media por filamento intraluminal, se les ocasionó una lesión focal en el hemisferio derecho. Posteriormente se evaluó el hemisferio contralateral, marcando la población neuronal con el anticuerpo monoclonal anti-NeuN. Se definieron cinco grupos de evaluación: uno de control, 24 h, 96 h, 10 días y 20 días. Se evaluaron los cambios neuronales exofocales después de la lesión con base en la observación de los cambios en la inmunoreactividad de las neuronas al NeuN.

Resultados: Se redujo la inmunoreactividad en la corteza contralateral a la lesión. Este fenómeno fue más notable en las capas supragranulares después de 24 h post isquemia. Después de 96 h hubo una disminución generalizada de la inmmunoreactivity en las capas supra e infragranulares. A los 10 y 20 días, el tejido recobró alguna inmunoreactividad NeuN, estos cambios se dieron en la capa VI.

Conclusiones: Los cambios inmunorreactivos a NeuN apoyan el proceso de diasquisis interhemisférica. Los cambios en la inmunorreactividad podrían indicar estrés metabólico secundario a la interrupción en la conectividad con el sitio de la lesión.

Corresponding author:
César Augusto Arango Dávila. Av. Simón Bolívar. Cra 98 # 18-49 Cali, Colombia. Tel: +57 2 331 9090, Fax: + 57 2 5551586. E-mail: arangodavila@gmail.com.

Introduction

Cerebrovascular disease (CVD) is a public health problem in the world. It is the leading cause of permanent disability in adults and the third leading cause of death in developed countries[1]. One out of six people suffers stroke (CVA), resulting from CVD during their lifetime, and about 30% have severe or permanent dysfunctions that make them dependent on others. According to the compromised cerebral territory, alterations can be motor, sensory, or cognitive mnemonic, among others, although generally there is a mix commitment[2]. Alterations may change over time due to exo-focal changes from injury caused by a plasticity process of the nervous system called diaschisis[3-5].

Strokes can be divided into ischemic, by decreased blood flow in specific brain area; or hemorrhagic, due to intracranial arterial rupture. Clinical practice says that 85% of strokes are ischemic; one of the most common is focal ischemia by occlusion of the middle cerebral artery (MCA)[6].

Cerebral ischemia is a pathophysiological condition characterized by transient or permanent, focal or global decrease of blood flow in the brain, which causes different patterns of tissue dysfunction according to the severity and duration of the ischemia[7]. When the flow of oxygen and glucose falls below 10 mL/100g/min, there is loss of function of the cell membrane by metabolic depletion of ATP pumps, the ion potential is lost, and cell swelling occurs due to increased intracellular sodium, leading in minutes to tissue necrosis and triggering inflammatory processes[8]. There is also an increase in the electrical activity of cell membranes at the edge of the focus, due to increased extracellular glutamate and potassium. This increases the excitability in cells, increases metabolic rate, and leads to the growth of the infarcted area. The directly affected area is called an ischemic focus, and it is the region showing the first consequences of the lesion[9-10].

Next to the ischemic core, it is established the penumbra, an area with low perfusion in which a residual flow persists, able to keep alive neurons. However, these neurons are dysfunctional due to metabolic stress. Because necrosis has not been established in the penumbra, it is an area susceptible of recovery; however, if an adequate reperfusion is not present, necrosis and stabilization of clinical deficit occur[7-11]. The penumbra is one of the therapeutic targets to reduce the consequences of ischemia, since it is where the needed angiogenesis processes are triggered to restore blood flow, and apparently, to stimulate endogenous neurogenesis[12]. However, there are zones of the penumbra area that are not restored, and that despite normalization of blood flow, undergo apoptotic cell death, days or months after the ischemia[13].

The ischemic cascade not only affects the focus and penumbra, but also exo-focal areas ipsilateral and contralateral to the lesion, and other structures such as the cerebellum, the thalamus, the substantia nigra, and the hippocampus. These areas and structures are disrupted due to their anatomical or functional connections with the ischemic focus and the penumbra area[14-16]. This phenomenon is not only related to pathological processes, but also with the reorganization of the nervous system and functional recovery[17], and it is seen in other disorders such as tumors, epilepsy and traumatic injuries[18].

There is evidence of transcallosal or contralateral diaschisis, due to the finding of bilateral brain disorders after unilateral lesions. Taking as stimulus the injury, the reorganization of the unaffected hemisphere is presented by disinhibition of commissural pathways and the relative increase of subcortical neuronal excitability, especially thalamic, probably as a resource to counteract the loss of contralateral function[19-20]. In this sense, the contralateral exo-focal alterations may be associated with brain plasticity, mechanisms of neuronal reorganization, and compensatory and adaptive processes[21-25]. It has been identified that, if it is performed ablation of the corpus callosum, the contralateral effects are reduced or do not occur, and that these changes create tolerance to future ischemic events[24-26].

In this aspect, it has been described the activation of over 80 genes in the ipsilateral and contralateral cerebral cortex, hippocampus, striatum and thalamus, which relate to circulatory and metabolic modifications[27]. It has also been shown dendritic arborization and axonal growth in the contralateral hemisphere, expression of neurotrophins with motor and sensory recovery, related to the organization of the pyramidal system after ischemia[28]. In addition, there is evidence of neurogenesis and synaptogenesis processes in some areas, and adaptive changes in the entorhinal cortex, among others[24-25,29].

Although it is not clear the mechanism of transcortical diaschisis[30], clinically, it is known that the contralateral alterations contribute to neurological deficit and heterogeneity of signs and symptoms in the mid and long term, such as cognitive deficits, loss of memory, aphasia and episodic amnesia[31-32]. However, few proposals are known to address this aspect of the cerebral damage[2-4]. From the above, it is concluded that it is important to study the contralateral hemisphere after ischemic brain injury as a contribution to theories of recovery and reorganization of brain functions. This is of clinical interest because, often, dysfunction and recovery are not directly related to the area and size of the lesion[32]. Learning about the pathophysiology of ischemia and transcortical changes is fundamental to understand the restoration processes of the systems affected by the injury, and consequently, to determine the most effective type of treatment.

The aim of this research was the descriptive characterization of contralateral cortical changes in an experimental model of focal cerebral ischemia to get evidence of cell reorganization, in order to understand the pathophysiological changes. It was used a monoclonal antibody anti-NeuN, specific for neurons (Neuronal Nuclei MAB377), with which, besides the nucleus, it is possible to mark the soma and the proximal processes of neurons. The marking process does not include glial cells or vascular components[34], unlike conventional histological methods that can eventually produce "noise" when observing cells. Within the nucleus, NeuN reactivity is mainly found in areas where there is low chromatin density, and it is largely excluded from areas containing densely packed DNA. The enzymatic dephosphorylation suppresses the immunoreactivity to NeuN, confirming that this is indeed a phosphoprotein, and it states that the antibody binding depends on the phosphorylation state[9]. It is clear that protein phosphorylation circumstances are significantly altered in ischemia. The NeuN has been identified with the Fox-3 protein, which like other proteins of this type, regulates mRNA splicing[35].

In pathological conditions such as cerebral ischemia, the decrease in quantifying NeuN cells may indicate neuronal loss due to necrosis[34]. In addition, it has been described a correlation among apoptotic cells, positive tunnel and immunoreactivity to NeuN with apoptotic cell death[36]. However, it has been established that the decrease in immunoreactivity to NeuN may be related to protein depletion or reduced immunoreactivity; changes that are not necessarily related to cell death, but with cellular metabolic stress[37]. If we consider that the transcallosal diaschisis corresponds to a phenomenon of cellular stress, assessing NeuN immunoreactivity in the cortex contralateral to the lesion can become a sensitive marker of exo-focal phenomena.

Materials and Methods

Twenty eight Wistar adult male rats were operated. The specimens weighted between 240 and 320 g (average 280 g), and were aged between 120 and 180 days. It was followed the protocol to avoid unnecessary suffering.

Microsurgical procedure

After a fasting period of 6 h, the experimental specimens were anesthetized with intraperitoneal ketamine hydrochloride (40 mg/kg) and atropine (0.1 mg/kg). The cerebral ischemia technique applied in this paper was developed by Zea *et al.*[38], and perfected by Belayeb *et al*[39]. Under a surgical microscope, a longitudinal incision was made in the midline of the neck. The common carotid artery and the external carotid artery (ECA) were located and dissected, being the latter cut, thus leaving free the ECA and linked at its end. A 4-0 nylon monofilament segment of 3.5 cm in length was introduced by the bottom of the corresponding ECA sac to a length of 17.5 mm and then fixed. After 90 min, the nylon was pulled through the surgical wound, and its end was left housed at the bottom of the ECA sac to start reperfusion. The four specimens of the control group underwent the described procedure, except that the 4-0 nylon monofilament was not introduced until the level of the middle cerebral artery (MCA). During the surgical procedure, the cardiorespiratory function and the rectal temperature were regularly evaluated. Temperature was maintained at 37° C by a heat lamp. Five groups were established, one control group with four specimens, and the remaining four with survival times at 24 h, 96 h, 10 days and 20 days; each one with six specimens.

Immunohistochemistry

At the corresponding time, the specimens of each group were anesthetized and trans-cardially perfused through the left ventricle with 200 mL of 0.9% saline, followed by 200 mL of a mixture of paraformaldehyde (4.0%), lysine and sodium periodate in 0.1 M phosphate buffer (pH 7.4). Then, the brains were removed from the braincase and left in the fixative. Later, 10 coronal sections of 30 μm thickness were performed in the vibratome of liquid medium; the cuts were made in accordance with the coordinates of the Atlas of Paxinos and Watson[40] in a sequential anteroposterior way, starting from the interaural point 10.7 with bregma 1.7, each mm to the interaural 3.7 point with bregma -5.3. The sections were incubated with the primary antibody NeuN (Neuronal Nuclei - MAB377 Chemicon) for 8 h; then, they were washed in phosphate buffer 10 mM (pH 7.4) and made react with secondary antibody using the avidin biotin kit (Vectastain), and then were revealed using diamino benzidine contrasted with nickel (ABC kit vector laboratories). The cuts, selected of the samples as control for the immunohistochemical process, suffered the same procedure described above, except for incubation with the primary antibody.

The cuts of the different experiment times were stained with 2,3,5-Triphenyltetrazolium chloride (TTC) to observe the infarcted area; for this type of vital staining, one of the subjects in each group was not perfused, it was deeply anaesthetized, and it was decapitated; then, the brain was rapidly removed from the braincase. The brains were cut coronally every 2 mm from the frontal pole using a brain matrix (Stoelting). The brain sections were immersed in a saline phosphate buffer solution (PBS) 0.1M pH 7.2 containing 0.5% TTC during 30 min at 37° C. Finally, the stained sections were fixed in 4% formalin and kept at 4° C. Six sections per brain were photographed in their anterior and posterior aspects, and the ischemic areas were manually measured in a blind way. Morphometric and volumetric analysis implied to correct the error caused by edema, for which it was used the formula [(normal hemisphere area / infarcted hemisphere area) x infarct area][41]. All experimental specimens showed lesion in an area proportional to approximate coronal section in Bregma 2.04 mm of 39.4 ± 4.2%. There were no major differences among the specimens, and variability in size among the groups was very low. The volume of ischemic damage was calculated by integrating the area of the corrected lesion of the different coronal cuts with the anteroposterior coordinate[42]; thus, the result is expressed in cubic millimeters of lesion; it was observed a substantial homogeneity in the different specimens 162.5 ± 17.5 mm^3.

The evaluation of the processed cuts by NeuN immunohistochemistry was performed in a qualitatively semistructured way, since each of the researchers independently examined the plates at 10x and 40x with specimen cuts in the control group and the experimental ones, focusing on cuts corresponding to the frontoparietal cortex, this was systematically done following an encoded sequence with a previously established order, and without knowledge of the type of the specimen that was being observed. Prior to this procedure, it was performed an assessment and training of all researchers to ensure the greatest possible objectivity in the observations. It began with a global observation of the sheets at 10x, and then a review sheet by sheet at 40x from I to VI, paying attention to the degree of immunoreactivity of the neurons to NeuN (immunoreactivity intensity similar to control v/s immunoreactivity intensity lower than control - neuronal morphology similar to control v/s neuronal morphology different to control). Each of the descriptions was recorded in the same type of format to be collected by the principal investigator, images for analysis of results were selected when the coincidence of the observation was 80% or more. To obtain the pictures, researchers were asked to select three patterns considered as representative of each change observed by them as relevant; from this selection, in turn, the lead researcher took random pictures to illustrate the publication, and for the final analysis.

On the other hand, changes were quantified in the number of positive NeuN cells in lamina VI: three areas of similar size for each of the specimens were demarcated, photographed and processed in black and white to 50% in the image format of the program World of Office, the images were printed and neurons

were manually counted. Each of researchers sent the results and they were integrated by a simple summation to define the proportion of neurons relative to the control.

Ethics

The state of control of the 28 subjects in all phases of the research was conducted using a monitoring protocol that establishes criteria to avoid unnecessary suffering, especially after surgery. The experimental protocol was assessed and approved by the ethics committee of the Universidad Libre (Cali, Colombia), to comply with the current regulations of the European Economic Community for the use and care of animals used for experimental and other scientific purposes (Strasbourg, 15 June of 2006), and the Colombian law (Law 84 of 1989 and Resolution No. 8430 of the Ministry of Health, 1993) on ethics, care and control of animals for experimental purposes.

Results

There were processed from the control group, six specimens. From the experimental groups of 24 h, 4 specimens; 96 h, 5 specimens; 10 days, five specimens; and 20 days, 4 specimens.

Mortality in rats was 21%, distributed by groups as follows: Control, 0%; 24 h, 7.1%; 96 h, 3.6%; 10 h, 3.6%, and, 20 days, 7.1%.

In this study the changes in the region contralateral to the lesion of the frontoparietal cerebral cortex were evaluated qualitatively. The results were obtained based on the data provided by the experimental groups, confronted with the control group, and descriptive analysis of changes in the immunoreactivity of neurons to NeuN. In all specimens subjected to ischemia and reperfusion, it was observed frontoparietal lesion in the territory of the MCA ipsilateral to the lesion, which includes loss of anatomical and histological features with degrees of neuronal damage ranging from obvious injury at 24 h, up to total loss of tissue with the formation of cystic areas (Fig. 1), as we have reported in other studies[4,9,33].

At 24 h, in the contralateral area, there were changes in neurons

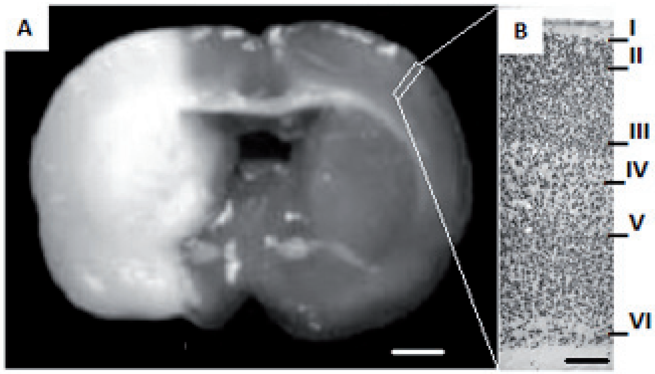

Figure 1. Coronal section stained with TTC after focal cerebral ischemia and reperfusion with intraluminal filament technique. In A, the area of injury, including the cerebral cortex and the basal ganglia, is observed pale. On the right, it is observed the cerebral cortex enlarged at 10x, where NeuN labeled neurons organized into sheets can be differentiated. Descriptive observations of all specimens were taken from the approximate area indicated in the image corresponding to a coronal cut in Bregma 2.04 mm. (A bar= 2 mm, B bar= 250 μm).

with decreased immunoreactivity to NeuN, compared with controls (Fig. 2 C and D); these changes are remarkable in supragranular layers. At 96 h post-ischemia, in the cortex of the hemisphere contralateral to the lesion, it is characteristic the decrease of cell immunoreactivity in supra and infragranular layers. In addition, it is appreciated cell nuclei with fractionation and consolidation with morphologic appearance resembling the nuclear fragmentation observed in apoptotic cell death (Fig. 2E and F). At 96 h, it can be seen transition areas from an organized tissue, to one where the cyto-architecture appearance is lost, especially in layer VI, which includes areas of disruption in the continuity of the layer and apparent neuronal loss (Fig. 3C).

Ten days post-ischemia in the contralateral hemisphere to the lesion, it can be seen unchanged cortical layers in their immunoreactivity, when they are compared with control (Fig. 2 G and H). At 20 days post-ischemia, the finding is indistinguishable from those found in the specimens at 10 days, which consist in an immunoreactivity to NeuN similar to controls; different from that observed in samples at 24 and 96 h.

An interesting observation was that 60% of subjects had alterations in the morphology of the layer VI (Fig. 3), changes that were detected in a more evident way at 96 h post-ischemia (Fig. 3 C), and that continued being seen until 10 days (Fig 3 D.) and 20 days (Fig 3 E.). It was observed in this layer impairing of the tissue cyto-architecture and a progressive decrease and loss of neuronal

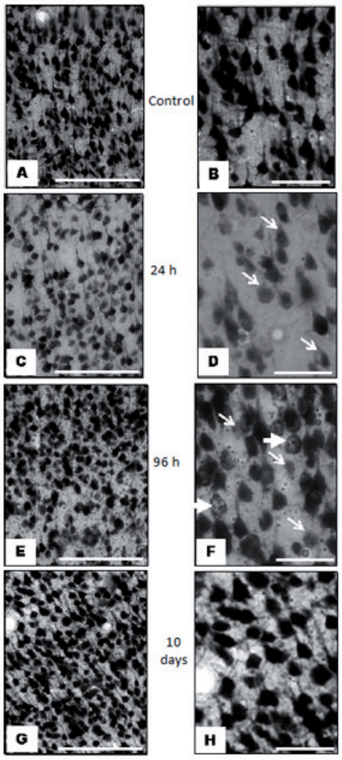

Figure 2. Images representative of immunoreactivity to NeuN in the frontoparietal cortex contralateral to ischemic injury, in an experimental model of focal cerebral ischemia of the middle cerebral artery. A and B, control images of the infra-granular zone at level of layer V, where it is observed appropriate immunoreactivity at 24 h. C and D, decreased immunoreactivity at 24 h in the supra-granular cerebral cortex, at level of layer III (arrows in D). In E and F, at 96 h, it is observed in the infra-granular cerebral cortex, at level of Layer V, neurons and cell nuclei with heterogeneous immunoreactivity (the thin arrows indicate neurons with decreased immunoreactivity; the thick arrows, neurons with split and consolidated nuclei). G and H, immunoreactivity at 10 days, supra-granular at level of layer III similar to control. Descriptive comments of all specimens correspond to coronal sections in Bregma 1.80 mm to 2.16 mm, middorsal approach 3.85 to 4.8 mm, and dorsoventral 2 to 3 mm. (First column bar= 250 μm, second column bar= 25 μm).

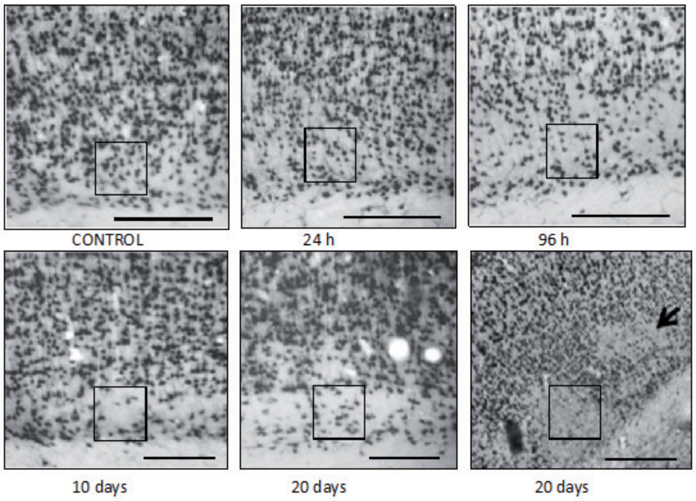

Figure 3. Images representative of immunoreactivity to NeuN in layer VI of frontoparietal cortex contralateral to ischemic injury, in an experimental model of focal cerebral ischemia of the middle cerebral artery. A control; B to F, changes in layer VI remarkable in the course of time; it is indicated, with boxes, the areas with altered immunoreactivity to NeuN, and apparent decrease of cellularity in F with panoramic view at 10x, it is seen loss in patches in some sectors of the layer VI. The number of immunoreactive neurons decreased by 4.5% at 24 h, 22.7% at 96 h, 36.4% at 10 days, and 40.9% at 20 days. (Bar A to E= 300 µm, F= 400 µm).

immunoreactivity: relative to quantification of neurons in the controls that we defined as a proportion of 100%, the number of immunoreactive neurons decreased to 95.5% and 77.3% at 24 and 96 h, respectively; at 10 and 20 days, the amount of immunoreactive neurons corresponds to 63.6% and 59.1% respectively. Panoramic view at 10x magnification, it is seen the loss in patches in some sectors of the layer VI (Fig. 3F).

Discussion

The analysis of changes in neurons of the hemisphere contralateral to focal ischemic injury has been little evaluated by using specific markers for neuronal population[43-45]. Nagasawa and Kogure[46] found no histological abnormalities in the contralateral hemisphere after focal cerebral ischemia in an experimental model similar to ours. They used Nissl staining and hematoxylin, which are not specific for neuronal population[42]. On the other hand, it has been found differences in susceptibility to stroke among different strains of rats[47], which could be related to the heterogeneity of the results of various investigations, especially regarding contralateral exo-focal changes.

In this study, the intensity of the immunoreactivity was considered as a pathophysiological measure of ischemic brain damage, as it has been noted in other works[37, 48]. The most significant changes are in dorsolateral areas in both hemispheres, regions that suffer in this model increased ipsilateral neuronal loss and changes in immunoreactivity of neurons in the contralateral cortex. In the frontoparietal region ipsilateral to the lesion, varying degrees of neuronal damage were observed, ranging from obvious necrosis at 24 h, up to total loss of tissue with formation of cystic areas, as it has been reported in other studies[4,9,49].

With regard to exo-focal changes in cortex contralateral to lesion, our study has three aspects to highlight. First, at 24 h after ischemia, decreased immunoreactivity to NeuN is evidenced in the supra-granular layers of the frontoparietal cortex contralateral to injury; at 96 h, changes are evident in both supra-granular and infra-granular layers. It is noticeable the fact that some neurons at 96 h exhibit immunoreactivity that account for an apparent fragmentation of nuclei, hitherto not described with NeuN (Fig. 2 F). It is important to note that previous studies have established a co-localization of neurons with reduced immunoreactivity to NeuN, and positive tunnel test[37].

Second, 10 and 20 days after ischemia, the immunoreactivity to NeuN, previously attenuated, is reset in supra-granular and infra-granular areas, but an alteration of residual appearance in layer VI is evident (Fig. 3), because it remains from 96 h until 20 days posterior to the ischemic injury. Previously, there were described processes of cortical plasticity related with thalamus-cortical connections, since the response of the cortex could be related to afferents coming from the thalamus to the layer VI[20,50]. Then, it arises the question that apart from the signals of de-afferentation of the ischemic focus through the corpus callosum, thalamic afferents would have an effect on cortical reorganization[17,19].

Third, it has been noted that the mechanisms of transcallosal exo-focal changes involve commissural connections and synapsis modulation[4,15,51]. Hyper-excitability has been observed in the contralateral hemisphere, as well as changes in metabolism that could be related to these transcallosal exo-focal changes[52]. The decrease in the activity of GABAergic neurons in the injured hemisphere and increased glutamatergic activity caused by ischemia can translate into overdrive in the not-injured hemisphere[26]. In this way, it could be explained the reorganization of motor functions after a CVA[51,52]. It has been described that in rats subjected to focal ischemia, the motor cortex in the non-ischemic hemisphere undergoes a process of reduction of dendritic spines, followed by arborization in the infra-granular layers. At the peak of dendritic growth, 18 days after ischemia, the synaptic density in infra-granular layers and spine density is normal[16].

This is a descriptive study of immunoreactivity to NeuN in the cortex contralateral to lesion. While it allows us to observe a sequence of events related to the intensity of the immunohistochemical reaction and morphological findings, the results should be expanded in subsequent studies, in which optical densities and quantification of neurons can be assessed. Other studies are also missing, that expand our insight about the role of NeuN antibody as a marker of cellular stress and potential marker of cellular death.

Conclusion

This study descriptively shows changes in cerebral cortex contralateral to lesion after transient focal ischemia in the territory of the middle cerebral artery in an experimental model in rats. Immunoreactivity to NeuN is taken as the primary means of analysis. So far, no studies are known of transcallosal diaschisis with observation of such marker. It was observed specific variation in the intensity of the immunoreactivity to NeuN as a function of time from 24 h to 20 days, showing a sub-chronic response in supra-granular areas, and then in infra-granular ones. It is remarkable to observe changes in layer VI that remain from 96 h up to 20 days. In functional terms, the significance of changes in neuronal immunoreactivity to NeuN could be associated with adaptive processes of contralateral cell reorganization after

ischemic event, in which cortico-cortical and cortico-thalamic projections would participate, as well as local circuits.

Financing:
Pontificia Universidad Javeriana, Cali, Colombia (Code 00003579) Neuroscience and Neuropsychology Line from the Group of Measurement and Psychological Assessment. Project "Reorganization on exo-focal regions (substantia nigra reticulata and dendritic fascicles in the frontoparietal cortex) for experimental transient focal ischemic injury in rats". For the information processing and work synthesis, we had support from resources of Valle del Lili Foundation and ICESI University of Cali, Colombia. We also had resources from the Innovation and Science Foundation for Colombia FICC through the Center for Innovation Research and development IDRC Line of Research in Experimental Neurosciences, Project "Brain Injury and Neuro-rehabilitation", providing human talent and micro-photographic record.

Knowledge:
Thanks to the Animal Ethics Committee of Universidad Libre of Cali, which evaluated this work.

Conflict of interest:
The authors state no conflict of interest.

References

1. Mozaffarian D. Heart disease and stroke statistics-2015 update: A report from the American Heart Association. Circulation. 2015; 131: e29–e32.

2. Caoa M, Ferraria M, PatellaaÇ R, Marrab C, Rasura M. Neuropsychological findings in young-adult stroke patients. Arch Clin Neuropsychol. 2007; 22: 133-42.

3. Bonilha L, Nesland T, Rorden C, Fillmore P, Ratnayake RP, Fridriksson J. Mapping remote subcortical ramifications of injury after ischemic strokes. Behavioural Neurology. 2014; 21: 53–80.

4. Arango-Dávila CA, Escobar-Betancourt MI, Buriticá E, Pimienta H. Cambios exofocales en la isquemia cerebral focal experimental: una visión experimental y su correlato clínico. Colomb Med. 2008; 39: 85–94.

5. Bütefisch CM, Kleiser R, Seitz RJ. Post-lesional cerebral reorganization: evidence from functional neuroimaging and transcranial magnetic stimulation. J Physiol Paris. 2006; 99: 437–454.

6. Andersen KK, Olsen TS, Dehlendorff C, Kammersgaard LP. Hemorrhagic and ischemic strokes compared: stroke severity, mortality, and risk factors. Stroke. 2009; 40: 2068-72.

7. Dirnagl U. Pathobiology of injury after stroke: the neurovascular unit and beyond. Ann N.Y Acad Sci. 2012; 1268: 21–5.

8. Anderova M, Vorisek I, Pivonkova H, Benesova J, Vargova L, Cicanic M, *et al.* Cell death/proliferation and alterations in glial morphology contribute to changes in diffusivity in the rat hippocampus after hypoxia-ischemia. J Cereb Blood Flow Metab. 2011; 31: 894–907.

9. Arango-Dávila CA, Escobar-Betancourt M, Cardona-Gómez GP, Pimienta-Jiménez H. Fisiopatología de la isquemia cerebral focal: aspectos básicos y proyección a la clínica. Rev Neurol. 2004; 39: 156–65.

10. Kumar A, Aakriti, Supta V. A review on animal models of strokeAn update. Brain Res Bull. 2016; 122: 35–44.

11. Lo EH. A new penumbra: transitioning from injury into repair after stroke. Nat Med. 2008; 14: 497–500.

12. Ortega FJ, Jolkkonen J. Restorative therapies to enhance sensorimotor recovery following cerebral ischemia. Acta Neurobiologiae Experimentalis. 2013; 73(1): 66–78.

13. Broughton BRS, Reutens DC, Sobey CG. Apoptotic mechanisms after cerebral ischemia. Stroke. 2009; 40: e331–9.

14. Chen B, Schulz R, Bönstrup M, Hummel FC, Sedlacik J, Fiehler J, Gerloff C, Thomalla G. Structural plasticity of remote cortical brain regions is determined by connectivity to the primary lesion in subcortical stroke. J Cereb Blood Flow Metab. 2015; 35(9): 1507–14.

15. Reinecke S, Lutzenburg G, Hagemann G, Bruehl C, Neumann-Haefelin T, Witte OW. Electrophysiological transcortical diaschisis after middle cerebral artery occlusion (MCAO) in rats. Neurosci Lett. 1999; 261: 85–8.

16. Nudo RJ. Recovery after damage to motor cortical areas. Curr Opin Neurobiol. 1999; 9: 740–7.

17. Guzzetta A, Bonanni P, Biagi L, Tosetti M, Montanaro D, Guerrini R, *et al.* Reorganization of the somatonsensory system after early brain damage. Clin Neurophysiol. 2007; 118: 1110–21.

18. Gil JL. El enigma de la diásquisis. Rev Neurol. 2001;32:199–200.

19. Iizuka H, Sakatani K, Young W. Neural damage in the rat thalamus after cortical infarcts. Stroke. 1990; 21: 790–4.

20. Reuck J, Decoo D, Lemahieu I, Strijckmans K, Goethals P, Van Maele G. Ipsilateral thalamic diaschisis after middle cerebral artery infarction. J Neurol Sci. 1995; 134: 130–5.

21. Lotze M, Beutling W, Loibl M, Domin M, Platz T, Schminke U, Byblow WD. Contralesional motor cortex activation depends on ipsilesional corticospinal tract integrity in well-recovered subcortical stroke patients. Neurorehabil Neural Repair. 2012; 26(6): 594–603.

22. Takatsuru Y, Fukumoto D, Yoshitomo M, Nemoto T, Tsukada H, Nabekura J. Neuronal circuit remodeling in the contralateral cortical hemisphere during functional recovery from cerebral infarction. J Neuroscience. 2009; 29(32): 10081–6.

23. Hirouchi Y, Suzuki E, Mitsuoka C, Jin H, Kitajima S, Kohjimoto Y, *et al.* Neuroimaging and histopathological evaluation of delayed neurological damage produced by artificial occlusion of the middle cerebral artery in Cynomolgus monkeys: Establishment of a monkey model for delayed cerebral ischemia. Exp Toxicol Pathol. 2007; 59: 9–16.

24. González-Aguado E, Martí-Fábregas J, Martí-Vilalta JL. El fenómeno de la diásquisis en la enfermedad cerebral vascular. Rev Neurol. 2000; 30: 941–5.

25. Harrison TC, Silasi G, Boyd JD, Murphy TH. Displacement of sensory maps and disorganization of motor cortex after targeted stroke in mice. Stroke. 2013; 44(8): 2300–6.

26. Reggia JA. Neurocomputational model of the remote effects of focal brain damage. Med Eng Phys. 2004; 26: 711–22.

27. Zhao LR, Mattsson B, Johansson BB. Enviromental influence on brain derived neurotrophic factor messenger RNA expression after middle cerebral artery occlusion in spontaneously hypertensive rats. Neuroscience. 2000; 97: 177–84.

28. Keyvani K, Reinecke S, Abts HF, Paulus W, Witte OW. Suppresion of proteasome C2 contralateral to ischemic lesions in rat brain. Brain Res. 2000; 858: 386–92.

29. Sopala M, Frankiewicz T, Parsons C, Danysz W. Middle cerebral artery occlusion produces secondary, romete impairment in hippocampal plasticity of rats -involvement of N-methyl-D-aspartate receptors? Neurosci Lett. 2000; 281: 143–6.

30. Johnston DG, Denizet M, Mostany R, Portera-Cailliau C. Chronic in vivo imaging shows no evidence of dendritic plasticity or functional remapping in the contralesional cortex after stroke. Cerebral Cortex. 2013; 23(4): 751–62.

31. Lotze M, Beutling W, Loibl M, Domin M, Platz T, Schminke U, *et al*. Contralesional motor cortex activation depends on ipsilesional corticospinal tract integrity in well-recovered subcortical stroke patients. Neurorehabilitation Neural Repair. 2012; 26(6): 594–603.

32. Cramer SC, Bastings EP. Mapping clinically relevant plasticity after stroke. Neuropharmacology. 2000; 39: 842–51.

33. Arango-Dávila CA, Pimienta-Jiménez H, Escobar-Betancourt M. Depresión postisquemia cerebral: aproximación clínica y fisiopatológica. Rev Colomb Psiquiatr. 2000; 4: 321–44.

34. Wolf HK, Buslei R, Schmidt-Kastner R, Schmidt-Kastner PK, Pietsch T, Wiestler OD, *et al*. NeuN: A useful neuronal marker for diagnostic histopathology. J Histochem Cytochem. 1996; 44: 1167–71.

35. Lee JC, Ahn JH, Lee DH, Yan BC, Park JH, Kim IH, *et al*. Neuronal damage and gliosis in the somatosensory cortex induced by various durations of transient cerebral ischemia in gerbils. Brain Res. 2013; 1510: 78–88.

36. Morancho A, García-Bonilla L, Barceló V, Giralt D, Campos-Martorell M, Garcia S, *et al*. New method for focal transient cerebral ischaemia by distal compression of the middle cerebral artery. Neuropathol Appl Neurobiol. 2012; 38: 617–27.

37. Unal-Cevik I, Kilinç M, Gürsoy-Ozdemir Y, Gurer G, Dalkara T. Loss of NeuN immunoreactivity after cerebral ischemia does not indicate neuronal cell loss: a cautionary note. Brain Res. 2004; 23: 169–74.

38. Zea-Longa E, Weinstein PR, Carlson S, Cummings R. Reversible middle cerebral artery occlusion without craniectomy in rats. Stroke. 1989; 20: 84–91.

39. Belayeb L, Zhao W, Ginsberg D. Transient middle cerebral artery occlusion by intraluminal suture: II: Neurological deficit, pixel-based correlation of histopathology with local blood flow and glucose utilization. J Cereb Blood Flow Metab. 1997; 17: 1281–90.
40. Paxinos G, Watson C. The Rat Brain in Stereotaxic Coordinates. San Diego: Academic Press; 1986.

41. Leach M, Swam J, Eisenthal D, Dopson M, Nobbs M. BW619C89 a glutamato release inhibitor, protects against focal cerebral ischemic damage. Stroke. 1993; 24: 1063–7.

42. Osborne KA, Shigeno T, Balarsky AM, Ford I, McCulloch J, Teasdale GM, Graham DI. Quantitative assessment of early brain damage in a rat model of cerebral ischaemia. J Neurol Neurosurg Psychiatry. 1987; 50: 402–10.

43. Tamura A, Nakane M, Kuroiwa T, Nagaoka T, Sano K. Imaging of remote areas after focal cerebral ischemia. Int Congr Series. 2003; 1252: 321–7.

44. Grefkes C, Fink GR. Reorganization of cerebral networks after stroke: new insights from neuroimaging with connectivity approaches. Brain. 2011; 134: 1264–76.

45. Zepeda A, Vaca L, Arias C, Sengpiel F. Reorganization of visual cortical maps after focal ischemic lesions. J Cereb Blood Flow Metab. 2003; 23: 811–20.

46. Nagasawa H, Kogure K. Correlation between cerebral blood flow and histologic changes in a new rat model of middle cerebral artery occlusion. Stroke. 1989; 20: 1037–43.

47. Durukan A, Tatlisumak T. Acute ischemic stroke: Overview of major experimental rodent models, pathophysiology, and therapy of focal cerebral ischemia. Pharmacol Biochem Behav. 2007; 87: 179–97.

48. Hossmann KA. Experimental models for the investigation of brain ischemia. Cardiovasc Res. 1998; 39: 106–20.

49. Wegner S, Weber R, Ramos-Cabrer P, Uhlenkueken U, Sprenger C, Wiedermann D, *et al*. Temporal profile of T2-weighted MRI distinguisher between pannecrosis and selective neuronal death after transient focal cerebral ischemia in the rat. J Cereb Blood Flow Metab. 2006; 26: 38–47.

50. Rossini PM, Calautti C, Pauri F, Baron JC. Post-stroke plastic reorganization in the adult brain. Lancet Neurol. 2003; 2: 493–502.

51. Rehme AK, Fink GR, von Cramon DY, Grefkes C. The role of the contralesional motor cortex for motor recovery in the early days after stroke assessed with longitudinal FMRI. Cerebral Cortex. 2011; 21(4): 756–68.

52. Bütefisch CM, Kleiser R, Körber B, Müller K, Wittsack HJ, Hömberg V, Seitz RJ. Recruitment of contralesional motor cortex in stroke patients with recovery of hand function. Neurology. 2005; 64(6): 1067–9.
